# Experimental Analysis and Neural Network Modeling of the Rheological Behavior of Xanthan Gum and Its Derivatives

**DOI:** 10.3390/ma16072565

**Published:** 2023-03-23

**Authors:** Madiha Melha Yahoum, Selma Toumi, Salma Hentabli, Hichem Tahraoui, Sonia Lefnaoui, Abdelkader Hadjsadok, Abdeltif Amrane, Mohammed Kebir, Nassim Moula, Amin Aymen Assadi, Jie Zhang, Lotfi Mouni

**Affiliations:** 1 Materials and Environment Laboratory (LME), University Yahia Fares of Medea, Medea 26000, Algeria; 2 Faculty of Sciences, Nouveau Pole Urbain, University Yahia Fares of Medea, Medea 26000, Algeria; 3 Laboratory of Experimental Biology and Pharmacology (LBPE), University Yahia Fares of Medea, Medea 26000, Algeria; 4 Laboratoire de Génie des Procédés Chimiques, Department of Process Engineering, University of Ferhat Abbas, Setif 19000, Algeria; 5 Laboratory of Biomaterials and Transport Phenomena (LBMTP), University Yahia Fares of Medea, Medea 26000, Algeria; 6 Functional Analysis of Chemical Processes Laboratory, Chemical Engineering Department, Saad Dahlab University, PB 270, Blida 09000, Algeria; 7 Ecole Nationale Supérieure de Chimie de Rennes, Centre National de la Recherche Scientifique (CNRS), ISCR—UMR 6226, Université de Rennes, F-35000 Rennes, France; 8 Research Unit on Analysis and Technological Development in Environment (URADTE-CRAPC), BP 384, Bou-Ismail 42004, Algeria; 9 Fundamental and Applied Research in Animal and Health (FARAH), Department of Veterinary Management of Animal Resources, Faculty of Veterinary Medicine, University of Liege, 4000 Liege, Belgium; 10 College of Engineering, Imam Mohammad Ibn Saud Islamic University, IMSIU, Riyadh 11432, Saudi Arabia; 11 School of Engineering, Merz Court, Newcastle University, Newcastle upon Tyne NE1 7RU, UK; 12 Laboratory of Management and Valorization of Natural Resources and Quality Assurance, SNVST Faculty, Akli Mohand Oulhadj University, Bouira 10000, Algeria

**Keywords:** artificial neural networks, chemical modification, rheological behavior, xanthan gum

## Abstract

The main objective of this study was to create a mathematical tool that could be used with experimental data to predict the rheological flow behavior of functionalized xanthan gum according to the types of chemical groups grafted onto its backbone. Different rheological and physicochemical analyses were applied to assess six derivatives synthesized via the etherification of xanthan gum by hydrophobic benzylation with benzyl chloride and carboxymethylation with monochloroacetic acid at three (regent/polymer) ratios R equal to 2.4 and 6. Results from the FTIR study verified that xanthan gum had been modified. The degree of substitution (DS) values varying between 0.2 and 2.9 for carboxymethylxanthan gum derivatives were found to be higher than that of hydrophobically modified benzyl xanthan gum for which the DS ranged from 0.5 to 1. The molecular weights of all the derivatives were found to be less than that of xanthan gum for the two types of derivatives, decreasing further as the degree of substitution (DS) increased. However, the benzyl xanthan gum derivatives presented higher molecular weights varying between 1,373,146 (g/mol) and 1,262,227 (g/mol) than carboxymethylxanthan gum derivatives (1,326,722–1,015,544) (g/mol). A shear-thinning behavior was observed in the derivatives, and the derivatives’ viscosity was found to decrease with increasing DS. The second objective of this research was to create an ANN model to predict one of the rheological properties (the apparent viscosity). The significance of the ANN model (R^2^ = 0.99998 and MSE = 5.95 × 10^−3^) was validated by comparing experimental results with the predicted ones. The results showed that the model was an efficient tool for predicting rheological flow behavior.

## 1. Introduction

Polysaccharides are polymers composed of a long sequence of monosaccharide units linked together by glycosidic bonds [1]. These macromolecules are generally hydrophilic and water-soluble. They are extracted from different resources such as algae, plants, microorganisms, and animals [1,2]. Polysaccharides are considered the most abundant natural biopolymers; hence, they are stable, non-toxic, biodegradable, and biocompatible. These biopolymers are used in different formulations as natural stabilizers and emulsifiers due to their gelling properties, as they can increase the viscosity of the aqueous phase of the emulsions [2,3].

Xanthan gum (XG) is a widely applied polysaccharide in the cosmetic and pharmaceutical industries [4]. It is a biodegradable, anionic, hydrophilic biopolymer produced by *Xanthomonas campestris*. One of the most important characteristics of xanthan gum is its ability to thicken and viscosifying liquids. Therefore, it is widely used in different emulsions to improve their functionality [4,5].

Despite all of its advantages, there are some disadvantages to using XG in its native form. Therefore, chemical modifications are necessary to broaden its spectrum of application by improving its physicochemical properties. As the chemical structure of XG includes several hydroxyl and carboxyl groups, thus, there are a variety of chemical modification methods that can be employed, such as etherification, esterification, and amidation [4].

Among the various methods of chemical modification on polymers, carboxymethylation of biopolymers is widely applied. Different studies have illustrated that novel derivatives of xanthan gum were obtained by carboxyméthylation [4,6]. These derivatives showed improved water dispersion characteristics and flow properties. The chemical modification of this hydrophilic biopolymer by fixing hydrophobic groups provided the molecule with an amphiphilic character that enhanced its physicochemical properties like improving XG’s capacity for self-organization in an aqueous solution and also increasing its adsorption properties at the interfaces separating an aqueous liquid phase from a much less polar liquid phase [4]. On the other hand, benzylation has recently attracted considerable interest, and it is widely applied to biopolymers [7,8,9]. Benzylation reactions occur between benzyl chloride and the hydroxyl groups of a biopolymer, forming ether linkages that make it more compatible with non-polar matrices and decrease its hydrophilic nature [7].

Xanthan gum is widely employed in various applications due to its exceptional and distinctive rheological properties [6]. XG’s rheological behavior reveals the well-ordered conformation of XG and its consistent consequent intermolecular interactions in aqueous solutions [4]. Consequently, examining the rheological behavior of XG derivatives is of great interest due to their altered chemical structures and physicochemical properties. Thus, it is required to accurately investigate the rheological behavior of this biopolymer and its derivatives.

On the other hand, several studies have demonstrated that the rheological properties can be predicted using algorithmic mathematical models [10,11]. In order to be able to predict a rheological property of a biopolymer, it is possible to teach an algorithm to recognize certain characteristics that influence this property. Once trained, and the input data are processed by the model, the desired property can be predicted. To perform this training, it is necessary to provide the input data and the value of the corresponding property to the algorithmic model [10]. In this way, the algorithmic model will create different instructions on its own to associate inputs and outputs, which is known as machine learning [10].

One of the most applied machine learning models is the artificial neural network (ANN), which is a nonlinear system inspired by the biological neural system and has demonstrated its effectiveness and consistency in a variety of domains by determining the relationship between input and output datasets [10,12]. An ANN model is based on a series of nodes in which the input data evolves by forming successive layers composed of neurons. In each layer, and at each neuron a calculated parameter is chosen called “weight.” By varying these weights, the model gradually comprehends and predicts the output value [12].

The present work aimed to evaluate the effect of hydrophobic modification by benzylation and hydrophilic modification by carboxymethylation, on the flow properties of xanthan gum. Different derivatives with variable degrees of substitution were synthesized by a Williamson synthesis and the effect of concentration and degree of substitution on the flow of each type of modified polymer were studied.

According to our knowledge, no anterior works exist on the synthesis of benzyl xanthan gum derivatives. Additionally, the second point that makes the originality of this work is the fact that no research has opted before for the comparison of the flow profiles of modified xanthan solutions as a function of the nature of the grafted group onto its skeleton. In addition, the development of an artificial neural network model (ANN) that can accurately predict this rheological behavior makes this work even more distinguished because such a study has never been done before.

## 2. Materials and Methods

### 2.1. Materials

Xanthan gum (XG) was obtained from Saidal company (Medea, Algeria), while mono chloroacetic acid (MCAA), benzyl chloride (BCL), ethanol, sodium hydroxide, acetic acid, and the rest of the chemical reagents were purchased from Sigma-Aldrich (Hamburg, Germany).

### 2.2. Chemical Modification of Xanthan Gum

The chemical modification of Xanthan gum was similar to the methods previously reported by Ahuja et al. and Yahoum et al. [4,6]. Briefly, 5 g of xanthan was suspended in 100 mL of ethanol and continuously stirred for 40 min at room temperature. Then, 5 mL of sodium hydroxide (NaOH at 16N) was added to the mixture at a rate of 1 mL each 15 min under magnetic stirring. Afterward, a specific amount (Table 1) of monochloroacetic acid (MCAA) or Benzyl chloride (BCl), which corresponds to the (reagent/polymer) molar ratios (R) equal to 2, 4, or 6, was gradually added to the mixture. The mixture was then heated at 50 °C for 4 h. At the end of the reaction, the product was filtered by vacuum filtration, washed twice with ethanol (50 mL), neutralized (with acetic acid), and rinsed again with absolute ethanol. Finally, the obtained product was dried in a laboratory stove at 70 °C for 24 h and ground in glass mortar until a fine powder was obtained. The obtained products were composed of three carboxymethyl xanthan derivatives, CMX1, CMX2, and CMX3, and three benzyl xanthan gum derivatives, BXG1, BXG2, and BXG3.

### 2.3. Physicochemical Characterization of Derivatives

#### 2.3.1. Fourier Transform Infrared Spectroscopy (FTIR) Analysis

The IR spectra were recorded from the KBr pellets containing the powders of native xanthan and its derivatives (sample/KBr = 1:100) using a Bruker Tensor 27 spectrophotometer (FTIR) (Bruker, Bremen, Germany) in the range of 4000 cm^−1^–500 cm^−1^.

#### 2.3.2. The Substitution Degree Determination

(a) The substitution degree of the CMX derivatives

The degree of substitution (DS) for CMXs was determined using the method previously reported by Yahoum et al. [6]. A 0.2 g amount of CMX was dissolved in 50 mL of hydrochloric acid (HCl) at 0.1 mL/L; then, the prepared solution was titrated with 0.1 M of sodium hydroxide (NaOH). The same test was carried out on a blank solution with native XG. The value of the DS is calculated according to the following equations:

(1) $$\mathrm{DS}=\frac{933.A}{1000-80.A}$$

(2) $$A=\frac{{\left(V_{2}-V_{1}\right)}_{\mathrm{NaOH}}\times C_{\mathrm{NaOH}}}{m_{\mathrm{CMX}}}$$

where A is the amount of -CH_2_COOH and -CH_2_COONa per gram of sample, *V*_2_ is the volume of NaOH used during the titration of blank (mL), *V*_1_ is the volume of NaOH used during the titration of the samples (mL), *C*_NaOH_ is the concentration of NaOH (mol/L), and *m*_CMX_ is the mass of the sample (g).

(b) The substitution degree of the BX derivatives

The degree of substitution for BXs was determined by the method described by Ming-Fei et al. [7], as follows:

(3) $$\mathrm{DS}=\frac{A_{1}\left(-CH\;of\;the\;aromatic\;cycle\right)}{A_{2}\left(-CH\;of\;the\;methyl\;group\right)}$$

where *A* corresponds to the absorbance determined by FTIR, in which *A*_1_ is determined at 872 cm^−1^ and *A*_2_ between 2900 and 2800 cm^−1^.

#### 2.3.3. Molecular Weight Determination

To determine the molecular weight of the prepared derivatives, a viscosimetric method was employed [13]. An Ubbelohde viscometer DIN (SI Analytics, Hattenbergstraße, Germany) was used at 25 °C. Two different concentrations of native XG and its derivatives were prepared. A 0.27 g amount of XG, CMXs, or BXGs was added into a 200 mL solution of 0.01 M of NaCl, and then different solutions were prepared (at the concentrations of 0.1% and 0.075%), in which XG derivatives were in the dilute regime behaving as Newtonian fluids [13].

The molecular weight was determined by the intrinsic viscosity [η] according to the following equation (the equation of Mark–Houwink).

(4) $$\left[\eta \right]=kM^{\alpha }$$

The parameters of this equation were (at 25 °C); *k* was equal to 2.79 × 10^−3^ cm^3^/g, and *α* was equal to 1.2754.

### 2.4. Rheological Analysis

The rheological study was carried out with an MCR 302 Anton Paar Physica rheometer (Anton Paar, Ostfildern, Germany). The viscosity measurements were performed using a 25 cm in diameter plate–plate geometry with a gap of 1 mm. Solutions at 0.5% and 1% of native XG and its modified derivatives (CMX1, CMX2, CMX3, BXG1, BXG2, and BXG3) were prepared at 25 °C. These solutions were then used to determine the flow curves by the variation in the apparent viscosity (η_app_) as a function of the shear stress (γ) that ranged between 10^−3^ (s^−1^) to 10^3^ (s^−1^) [6].

### 2.5. Prediction of the Rheological Behavior Using Artificial Neural Network (ANN)

An artificial neural network (ANN) is an advanced, nonlinear, and empirical model comprising a multitude of units called neurons [14,15]. These neurons work together to form the network, and the functionality of the network is primarily determined by the connections between the neurons [16,17]. The neurons in an ANN are divided into three separate layers: the input layer, the output layer, and the hidden layer [18,19]. The input layer contains the same number of neurons as there are input variables, while the output layer has a corresponding number of neurons to the output variables [18]. In between the input and output layers is at least one hidden layer, the number of neurons, which is dependent on the specific algorithm used in the ANN [18].

In this study, the ANN model was used to predict the flow behavior of xanthan gum and its different derivatives (CMXs and BXGs) according to the physicochemical parameters. Four input parameters were considered: the type of sample (XG, BXG(s) or CMX(s)), the molecular weight of XG and the derivatives, the concentration of XG and derivatives (0.5% or 1%), the degree of substitution (DS), and the shear stress (γ). The variation in apparent viscosity (η_app_) was selected as the output parameter to be predicted by the ANN model. This model was then developed and validated using a database of currently obtained results. The architecture of the ANN model consisted of three layers: an input layer, a hidden layer, and an output layer. The training of the ANN model was carried out using 80% of the data set, and its performance was evaluated on 20% of the remaining dataset.

In order to assess the performance of the ANN, the mean square error (MSE) was calculated to demonstrate the statistical difference between the predicted and experimental values [12,15]. The accuracy of the developed model was assessed by the value of the correlation coefficient R^2^ [20,21,22,23,24,25].

When the ANN was developed, it was important to decide the number of neurons in the hidden layer [20]. The rate of combination of the system could be influenced by a couple of neurons in the hidden layer. On the other hand, the number of neurons in the hidden layer was acquired by experimentation wherein the minimum error between the experimental values and the predicted values was obtained. All input and output data were normalized between −1 and 1 [12,20].

### 2.6. Statistical Analysis

The statistical analysis was exploited by ANOVA using Tukey’s multiple comparison test. All experiments were repeated in triplicate, and *p* < 0.05 was considered statistically significant.

## 3. Results and Discussion

### 3.1. Physico-Chemical Characterization of Derivatives

#### 3.1.1. FTIR Analysis

**Figure 1:**
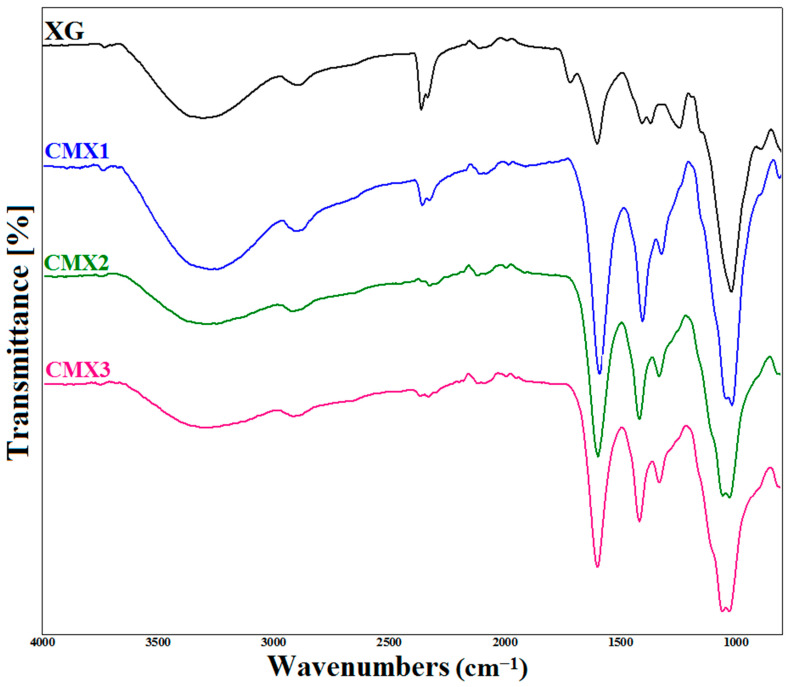
FTIR spectra of native and carboxymethyl derivatives of xanthan gum (CMXs).

Figure 1 presents the results of the FTIR analysis in which it was revealed that XG was successfully modified. All of the characteristic peaks of native XG were observed, in particular at 1022.36 cm^−1^ for the ether function, 1248.13 cm^−1^ for the acetal function, 1407 cm^−1^ for the carboxyl function, and 1602.13 cm^−1^ for the carbonyls, as well as the peak at 1718.53 cm^−1^ for the CH_2_OCOCH_3_ ester function of the acetyl group. The peaks at 2800 and 3315 cm^−1^ correspond, respectively, to the -CH_2_ [26,27], and -OH bonds [6].

The results of the FTIR analysis of the CMXs confirmed the modification of the xanthan gum by the appearance of the peak around 1300 cm^−1^ relative to the elongation of the C-O-C bond. The increase in the intensity of the peaks around 1600 cm^−1^ for the CMX was noted, confirming the increase in the fraction of the -COOH group. The disappearance of the peak at 1700 cm^−1^ was the result of acetylation by the alkaline reaction [4,6,26].

**Figure 2:**
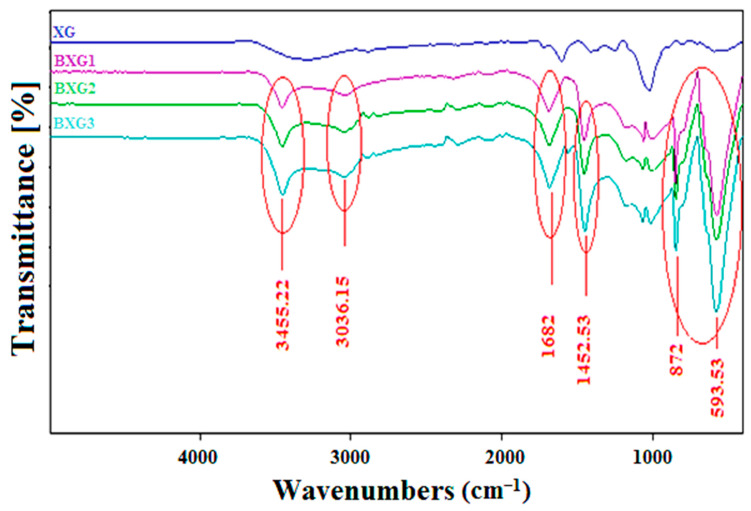
FTIR spectra of native and benzyl derivatives of xanthan gum (BXGs).

Figure 2 shows the FTIR spectra of native XG and its BXG derivatives. It can be noted that the peak at 1718 cm^−1^ disappeared on the spectra of the three derivatives BXG1, BXG2, and BXG3, due to the deacetylation caused by the alkaline reaction. It was also noted that new symmetrical peaks appeared at 598 and 872 cm^−1^ that corresponded to a mono-substituted aromatic cycle. Another characteristic peak appeared at 1110 cm^−1^ that was attributed to the ether bond [11]. The appearance of a new intense peak around 1500 cm^−1^ (1452.53 cm^−1^) for the derivatives BXG2 and BXG3 was linked to the aromatic cycle. The peak at 1682 cm^−1^ was attributed to the C=C functions of the aromatic ring, as well as the appearance of a peak at 1452 cm^−1^, which was attributed to the formation of the c-o bond (ether), which was highly intense for BXG3. Finally, a new elongation with a moderated intensity was observed at 3036 cm^−1^, which was attributed to an aromatic -CH. Its intensity increased with the degree of substitution, which confirmed the modification of xanthan gum. These results were similar to the ones obtained by Ahuja et al. and Yahoum et al. [4,6].

#### 3.1.2. Determination of the Degree of Substitution

**Figure 3:**
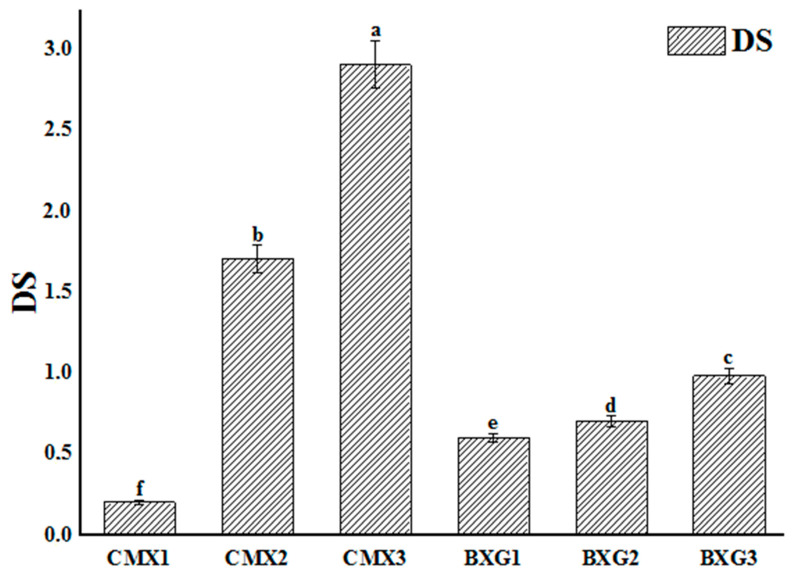
Results of determination of the degree of substitution (DS) of XG derivatives (CMXs and BXGs). Different superscript letters (a–f) indicate significant differences (*p* < 0.05).

The results of the degree of substitution are presented in Figure 3 below. It was noted that DS values varied between 0.2 and 2.9 for CMX derivatives and between 0.5 and 1 for BXG derivatives. These values were directly proportional to the molar ratio R. When the molar ratio increased, DS values also increased and reached 2.9 for the derivative CMX3 and 0.98 for the derivative BXG3. It is also clearly observed that DS values of benzyl derivatives were lower than the ones of CMXs (*p* < 0.05). This could be explained by the difference in affinity that existed between XG and the benzyl substitute, which was highly hydrophobic unlike the carboxymethyl group [4,7]. However, the carboxymethyl groups are more polar than the benzyl groups, and then their interactions with the hydrophilic alcoolate site are more effective because of the greater affinity between these polar entities.

#### 3.1.3. Determination of the Molecular Weight

**Figure 4:**
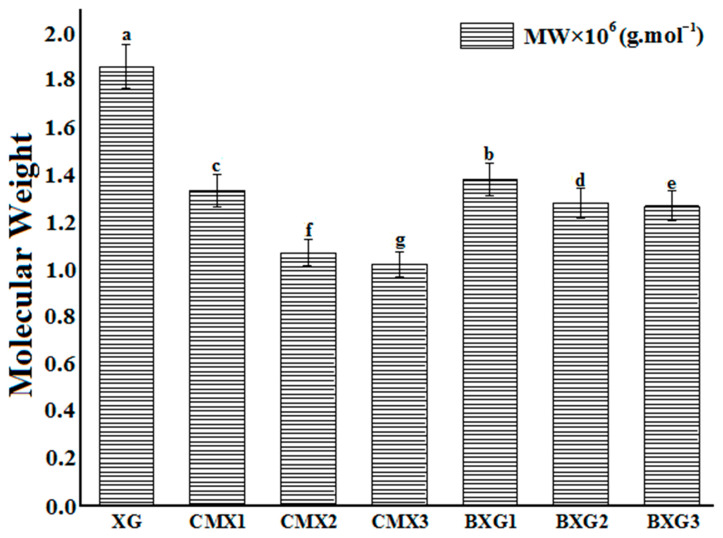
Results of determination of the molecular weight of XG and its derivatives (CMXs and BXGs). Different superscript letters (a–g) indicate significant differences (*p* < 0.05).

Figure 4 presents the obtained results of the molecular weight determination. It was noted that the molecular weights of all derivatives were lower than the molecular weight of native XG (*p* < 0.05). The obtained BXG derivatives presented higher molecular weights than CMX derivatives. It was also observed that when DS values increased, the values of the molecular weight decreased (*p* < 0.05). This effect was significantly more noticeable for CMX derivatives than for BXG derivatives (*p* < 0.05). In the case of CMX derivatives the molecular weight, a decrease is more significant because of the combined effect of the alkaline treatment necessary for the activation of the hydroxyl groups and the formation of alcoholates in addition to the effect of the reagent, which is of acidic nature and which contributes to the cleavage of the XG polymeric chain.

### 3.2. Rheological Analysis—Flow Curves

**Figure 5:**
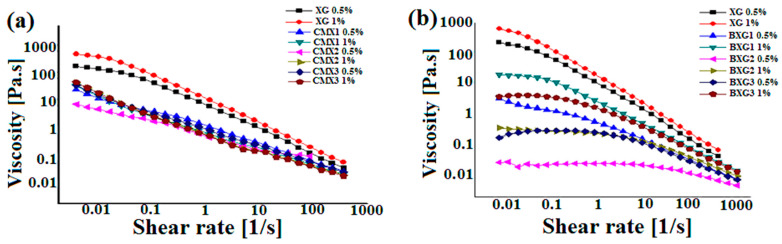
Flow curves of carboxymethylxanthan derivatives (CMXs) (**a**) and benzylxanthan derivatives (BXGs) (**b**) at concentrations of 0.5% and 1%.

Figure 5a illustrates the flow curves of XG and its derivatives (CMXs). It was clearly noted that the viscosity of XG and its derivatives decreased when the shear rates increased (*p* < 0.05), which validated that XG and its CMX derivatives presented a shear-thinning behavior. The viscosity of the modified derivatives was noticeably lower than that of XG (*p* < 0.05), particularly at high shear rates. Similar results were reported by Ahuja et al. and Yahoum et al. [4,6]. Yet, the effect of the degree of substitution was barely detectable on the viscosity, but practically, the viscosity decreases with the degree of substitution due to a decrease in molecular weight [6].

In addition, XG and CMXs had dissimilar flow curves. The flow curve of XG was characterized by two regions. The first region (I) was a Newtonian plateau at very low shear rates, while the second region (II) corresponded to the shear thinning flow due to the alignment of polymeric molecules within the flow field [28].

On the other hand, the flow curves of the modified derivatives exhibited three regions. The first region was characterized by a shear-thinning behavior, and the Newtonian plateau was not observed in that range, characteristic of yield stress fluids. At intermediate shear rates, the second region was noted where a short pseudo-Newtonian or equilibrium plateau was observed. Finally, the third region also exhibited a shear-thinning behavior.

Figure 5b represents the flow curves of XG and its derivatives (BXG). It is observed that XG and BXG derivatives had similar flow curves with two regions. At low shear rates, Region I was attributed to the Newtonian plateau, while shear-thinning behavior was observed in Region II. These results indicated that BXs exhibited a shear-thinning behavior with lower viscosity values than native XG due to the alkaline reaction during the modification process. Unlike the CMX derivatives, the effect of DS is significantly noticeable for the BXG derivatives.

For BXG1 and BXG2 derivatives, it was observed that the viscosity decreased when DS increased (*p* < 0.05). This result may be attributed to the fact that this can probably be related to the number of grafted hydrophobic groups compared to the preponderance of the hydrophilic chains of the molecule. In fact, in the BXG1 derivative, the interaction forces between the polymer chains are stronger than the hydrophobic interactions between the benzyl groups. However, in the BXG2 derivative, the number of benzyl groups increases, thus creating a greater number of hydrophobic microdomains within the network. This contributes to the unfolding of the chains by expelling anionic polar groups in an aqueous medium, and therefore, the electrostatic repulsion becomes preponderant, which will result in a decreased viscosity by the disentanglement of the polymeric network. These results are not totally in agreement with previous works related to the hydrophobic modification of xanthan by Sara et al. [26] and Toumi et al. [27,29] for kappa-carrageenan. This may be because, in these works, the length of the hydrophobic octyl moieties is more important than that of benzyl groups, which resulted in stronger associative forces that increased with increased DS.

Moreover, for the BXG3 derivative, higher viscosity is observed (*p* < 0.05) probably due to the greater number of hydrophobic groups. Therefore, in this case, the inter-intra-chain hydrophobic associative interactions between the benzyl groups become predominant compared to BXG2, thus increasing the entanglement within the network by compensating electrostatic repulsions, which created strong hydrophobic associations that made the rupture of the network very challenging [8]. Indeed, the xanthan gum chains become more rigid after grafting a hydrophobic group onto the backbone of the native XG. This result is in accordance with the findings of Lim et al. [28] for hydrophobically modified xanthan gum by chemical grafting of the octylamine chain.

The effect of the concentration on the viscosity for all of the derivatives was similar. It was found that the viscosity increased when the concentration increased (*p* < 0.05). Subsequently, when the concentration of the polymer increased, the entanglement of the polymeric chains within the network was notably stronger.

**Figure 6:**
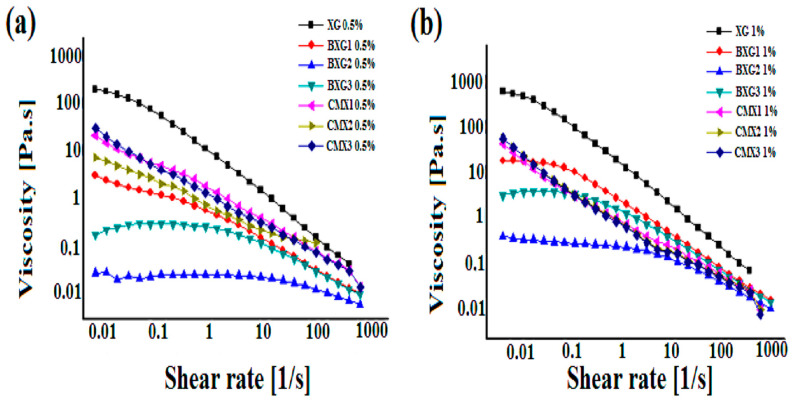
Flow curves of carboxymethylxanthan derivatives (CMXs) and benzylxanthan (BXGs) derivatives at concentrations of 0.5% (**a**) and 1% (**b**).

The rheological behavior of xanthan gum was noticeably affected by the nature of the derivatives (Figure 6). Grafting carboxymethyl groups onto the xanthan transformed the latter into a more viscous material at rest but at higher shear rates it exhibited more fluid characteristics [6]. These results are similar to those found by Badwaik et al. [30], where a pronounced pseudoplastic shear-thinning behavior was also noticed for the aqueous solution of carboxymethyl xanthan gum. This behavior was attributed to the presence of hydrogen bonds that stabilize the domains of linked polysaccharide chains at rest or low shear. Upon shearing, the hydrogen interactions are broken owing to the reduced aggregation, hence lowering the viscosity.

On the contrary, after the benzylation reaction, the skeleton of the xanthan gum has transformed the latter into an associative material with very interesting flow properties.

### 3.3. Prediction of Rheological Behavior by ANN

In this part, an artificial neural network model (ANN) was developed to predict one of the rheological characteristics (apparent viscosity) of xanthan gum and its derivatives. The developed ANN model used four independent variables as inputs (concentration of XG and its derivatives (0.5% or 1%), the molecular weight of XG and the derivatives, the degree of substitution (DS), and the shear rates) with a single output, which was the apparent viscosity (η_app_). The data necessary to develop the ANN model were obtained from the present experimental study. The database is composed of 405 different experimental data. Furthermore, 80% of the database is used for the training step, while 20% is for the testing step. Table 2 summarizes the obtained results, as well as the input and output variables implemented in the ANN model.

The performance of the ANN model was assessed with different statistical error measures. In the current study, the mean square error (MSE) and the coefficient of determination (R^2^) were used. Orange Python 3.26.1 software was used to calculate the predicted values for each experimental data. The obtained results of the prediction using the ANN model are presented in Table 2.

**Figure 7:**
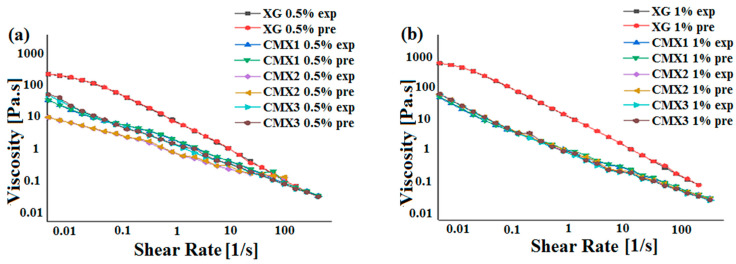
Flow curves of carboxymethylxanthan derivatives (CMXs) for the experimental (exp) and predicted (pre) results at the concentrations of 0.5% (**a**) and 1% (**b**).

**Figure 8:**
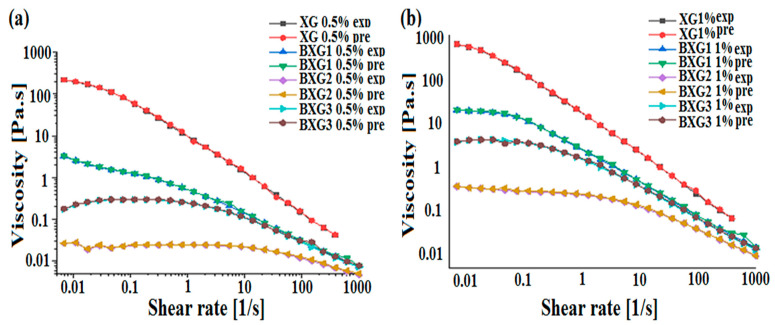
Flow curves of benzylxanthan derivatives (BXGs) for the experimental (exp) and predicted (pre) results at concentrations of 0.5% (**a**) and 1% (**b**).

The correlation between the experimental values and the predicted ANN output for the apparent viscosity after the training and testing steps are illustrated in Figure 7 and Figure 8. The obtained results illustrated a suitable correlation between the predicted values and the experimental ones, which demonstrates that the ANN is a well-fit model. The accuracy of this model was confirmed with an MSE equal to 5.95 × 10^−3^ and R^2^ equal to 0.99998.

Artificial neural network models are successfully applied in different domains to predict unknown parameters [12,20,31]. These models are widely explored in drug delivery to predict drug release from different drug delivery systems [31,32,33]. In our context, Razi et al. [10] developed an ANN model to evaluate the apparent viscosity of xanthan gum at different temperatures and concentrations. To our knowledge, this was the first study that applied an ANN model to predict the flow behavior of xanthan gum and its derivatives (carboxymethyl xanthan and benzylxanthan). It was concluded that this model is a great tool for the prediction of rheological behavior.

## 4. Conclusions

The present work consists of the synthesis and the development of different derivatives of xanthan gum by an etherification reaction with two reagents of different natures and at variable rations (R = 2.4.6). On the one hand, three carboxymethyl xanthan derivatives (CMX1, CMX2, and CMX3) were obtained by a reaction between xanthan gum and monochloroacetic acid (MCAA), and on the other hand, three benzyl xanthan derivatives (BXG1, BXG2, BXG3) were synthesized by reaction with benzyl chloride (BCl).

In the first part of this research, the six synthesized derivatives were subjected to various physicochemical tests making it possible to evaluate the effect of the substituent nature, the degree of substitution, and the concentration of biopolymers on the flow of the new derivatives in comparison with the native gum. The first step was the analysis by infrared spectroscopy (FTIR), after which the modification of the native xanthan was confirmed. This step was followed by the determination of the degree of substitution and the molecular weight, the results of which showed that the molecular weights of all the derivatives are lower than that of xanthan gum due to the alkaline treatment during the reaction. It has also been shown that the DS increases with the (reagent/polymer) ratio (R) but decreases with the molecular weight. However, it was found that this reduction is more marked and considerable for the carboxymethylated CMX derivatives compared to the benzylated derivatives where the reduction is less significant.

The rheological study revealed the shear-thinning behavior of native xanthan and all its CMX and BXG derivatives. On the other hand, the hydrophobically modified benzyl derivatives have higher viscosities than the carboxymethyl derivatives. Furthermore, the CMX derivatives present different flow profiles from those of XG and BXG, but which are much closer to yield stress fluids with the absence of the first Newtonian plateau.

In the second part of this study, an artificial intelligence-based model using an artificial neural network model (ANN) was developed to predict the apparent viscosity of native XG and its derivatives. The comparison of the experimental results with the predicted ones confirmed the relevance of the ANN model. It was concluded that this well-fit model is an excellent tool for the prediction of the rheological behavior of a biopolymer (R^2^ = 0.99998 and MSE = 5.95 × 10^−3^). This predictive model could be used to minimize several cost-effective and time-consuming experimental studies.

## Figures and Tables

**Table 1:** Amount of MCAA and BCL corresponding to different molar ratios.

R	2	4	6
qMCAA (g)	1.1	2.2	3.1
V_BCl_ (mL)	3.0	6.0	9.0

R: (reagent/polymer) molar ratio, qMCAA: the amount of MCAA, V_BCl_: the volume of BCl.

**Table 2:** Results of the ANN model prediction.

Inputs	Concentration of XG and Derivatives (0.5% or 1%), Molecular Weight, DS, and Shear Rate
Output	Apparent Viscosity
**Number of hidden layers** **Number of neurons in the hidden layers** **Number of learning data** **Maximum epochs** **MSE (training)** **R^2^ (training)** **MSE (testing)** **R^2^ (testing)** **Architecture of ANN**	02 150 405 12,780 8.43 × 10^−3^ 0.999953 5.95 × 10^−3^ 0.99998 4 × 150 × 2

MSE: mean square error, R^2^: coefficient of determination.

## Data Availability

The data presented in this study are available in the manuscript.
